# Immunoregulatory Effect of Koumine on Nonalcoholic Fatty Liver Disease Rats

**DOI:** 10.1155/2019/8325102

**Published:** 2019-02-17

**Authors:** Rongcai Yue, Guilin Jin, Shanshan Wei, Huihui Huang, Li Su, Chenxi Zhang, Ying Xu, Jian Yang, Ming Liu, Zhiyong Chu, Changxi Yu

**Affiliations:** ^1^School of Pharmacy, Fujian Medical University, Fuzhou, 350122 Fujian, China; ^2^Naval Medical Research Institute, Second Military Medical University, Shanghai 200433, China; ^3^School of Pharmacy, Second Military Medical University, Shanghai 200433, China

## Abstract

Nonalcoholic fatty liver disease (NAFLD) is the most common and important chronic liver disease all over the world. In the present study, we found that koumine, the main and active ingredient isolated from *Gelsemium elegans*, has the potential therapeutic effect on NAFLD rats by immunomodulatory activity. Koumine could significantly reduce the level of TG, TC, LDL-C, ALT, and AST in the serum of NAFLD rats and increase the level of HDL-C, reduce the liver index, and improve the adipose-like lesions of liver cells in NAFLD rats. Furthermore, treatment with koumine inhibited the severity of NAFLD. In addition, koumine-treated rats significantly increased the proportion of CD4^+^/CD8^+^ T cells and also decreased the percentages of Th1 and Th17 cells and increased Th2 and Treg cells in the liver. Moreover, koumine reduced the production and mRNA expression of proinflammatory cytokines *in vivo*. This result showed that koumine could effectively modulate different subtypes of helper T cells and prevent NAFLD. The present study revealed the novel immunomodulatory activity of koumine and highlighted the importance to further investigate the effects of koumine on hepatic manifestation of the metabolic syndrome.

## 1. Introduction

Nonalcoholic fatty liver disease (NAFLD) is the most common and important chronic liver disease all over the world [1, 2]. NAFLD is clinically divided into two forms: simple fatty liver (SFL) and nonalcoholic steatohepatitis (NASH), with NASH accounting for approximately half of all NAFLD cases. The probability of developing cirrhosis is 0.6%-3.0% in patients with SFL for 10-20 years and as high as a quarter in patients with NASH for 10-15 years. Approximately 1% of cirrhosis cases develop hepatocellular carcinoma each year [3–5]. Consequently, NAFLD represents a spectrum of disease ranging from hepatocellular steatosis through steatohepatitis to fibrosis and irreversible cirrhosis [6–8]. Although NAFL may occur in nonobese patients, most cases of NAFL are associated with obesity, type 2 diabetes mellitus, and hyperlipidaemia [9–11] and are accompanied by cardiovascular risk [12]. The prevalence of NAFLD has risen rapidly in parallel with the dramatic rise in obesity and diabetes, especially in the prevalence of obesity increases in adults and children [13, 14]. NAFLD is now recognized to represent the hepatic manifestation of the metabolic syndrome and is rapidly becoming one of the leading causes of liver disease in Western countries and China [15, 16]. The current medical treatment for NAFLD includes antioxidants, antiatherosclerotic drugs, hydroxymethylglutaryl coenzyme A reductase inhibitor, and angiotensin-II receptor antagonist [17–19]. However, since there is currently no specific treatment for NAFLD, it encourages the search for complementary and alternative treatments, such as medicinal medicine.

**Figure 1:**
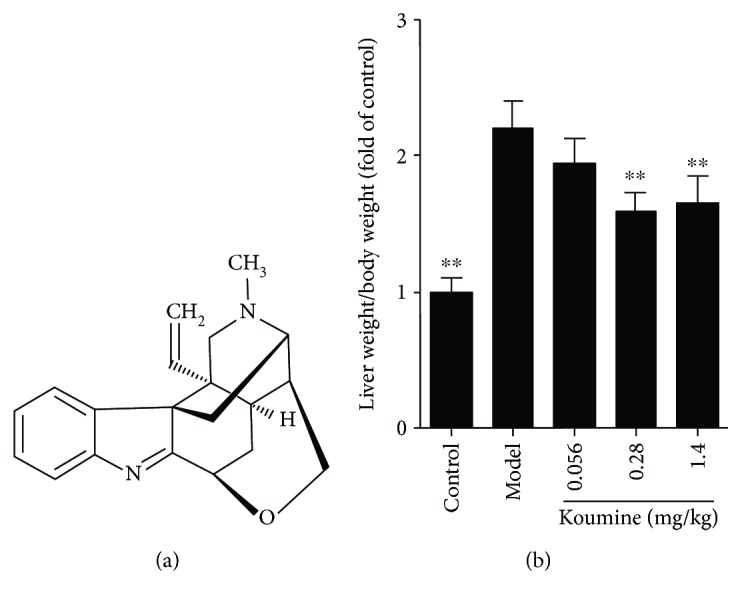
Chemical structures of koumine (a) and the effect of koumine on the liver index of NAFLD rats induced by fat diet. ^∗∗^*p* < 0.01 versus the model group.

Over the course of more than 2000 years, traditional Chinese medicine has identified and utilized many herbs for the treatment of various diseases. Herbal drugs obtained from the plant source are relatively safe and less expensive and possess good tolerability even at higher doses without severe side effects, which may have a profound impact on the treatment of NAFLD. Koumine (Figure 1(a)), an indole alkaloid isolated from *Gelsemium elegans*, has shown diverse pharmacological activities including antitumor, anti-inflammatory, and immunomodulatory activities [20, 21]. We recently reported the anti-inflammatory and immunoregulatory effect of koumine in a rheumatoid arthritis model [22–24] with a high-efficiency and low-toxicity feature, and next mechanism study found that koumine has the potential activated T cell regulation activity. Therefore, more immunomodulatory effects of koumine deserve further study.

The dysregulation of immune cells plays an important role in NAFLD, and several liver nonparenchymal cells including Kupffer cells, natural killer cells, and T lymphocytes are involved in the immune response of NAFLD morbidity process [25]. A longer adaptation process to injury and healing involves other cell types such as hepatocytes, hepatic stellate cells, and endothelial cells in addition to immune cells. Currently, effective treatment for NAFLD remains limited. In the present study, we investigated the therapeutic effect and mechanism of koumine on NAFLD. The result showed that koumine can effectively inhibit the development of NAFLD, decrease the clinical symptoms and inflammation, and reduce the infiltration of CD4^+^ T cells and activation in the liver. The present study will be very helpful for developing novel and effective strategies for NAFLD treatment.

## 2. Material and Methods

### 2.1. Rats

Male Sprague-Dawley rats (170-200 g) were purchased from Shanghai SLAC Laboratory Animal Co. Ltd. (Shanghai, China). All rats were housed in specific pathogen-free conditions (22°C, a 12 h light/dark cycle with the light cycle from 6:00 to 18:00 and the dark cycle from 18:00 to 6:00) with *ad libitum* access to standard laboratory chow. All animal experiments were approved by the ethics committee at Fujian Medical University (no. 2017-021), and the study was conducted in accordance with the guidelines published in the NIH Guide for the Care and Use of Laboratory Animals.

### 2.2. Drugs

Koumine (PubChem CID: 91895267; purity > 98.5%, HPLC; Figure 1(a)) was isolated from *Gelsemium elegans* Benth. via pH-zone-refining countercurrent chromatography, which has been described in our previous study (Su et al., 2011). Koumine was intraperitoneally injected at a dose of 1.4 mg/kg, 0.28 mg/kg, and 0.056 mg/kg dissolved in sterile physiological saline (0.9% NaCl).

### 2.3. Induction of NAFLD

After 1 week of adaptive feeding, 50 male SD rats were randomly divided into the control group (10) and the model group (40 rats). The control group was fed with ordinary feed, and the model group was fed a high-fat diet (cholesterol 1%, bile salt 0.1%, lard 10%, egg yolk powder and whole milk powder 5%, and the rest for ordinary feed). At the end of the sixteenth week, each group was given intraperitoneal injection of koumine or equal volume of saline once a day for two weeks. At the end of the eighteenth week, blood was collected from the abdominal aorta after anesthesia, and serum and liver tissue samples were collected.

### 2.4. Histopathology

Rats were anesthetized to obtain the liver tissue; the samples with 4% paraformaldehyde were perfused and fixed overnight. After routine operation, paraffin-embedded 5 *μ*m sections of the liver tissue were cut for HE staining and observed under the microscope (Olympus, Tokyo, Japan). Each histological sample was evaluated for the NAFLD activity score (NAS) as previously described [26].

### 2.5. Liver Index Measure

The liver was taken, cleaned with normal saline, dried with filter paper, and weighed. The liver index was calculated as liver weight compared to body mass.

### 2.6. Colorimetric Assay

The contents of serum total cholesterol (TC), triglyceride (TG), high-density lipoprotein (HDL), low-density lipoprotein (LDL), aspartate aminotransferase (AST), alanine aminotransferase (ALT), hepatic malondialdehyde (MDA), and nicotinamide adenine dinucleotide (NAD) were measured according to the kit instructions (Nanjing Jiancheng Bioengineering Institute).

### 2.7. Measurement of Cytokine Production

Serum and liver were collected to assay for cytokine levels. Serum was analyzed by a LEGENDplex™ kit (BioLegend, San Diego, CA) according to the manufacturer's protocol. Liver homogenate (10%, *w*/*v*) was prepared by homogenizing the liver tissue in PBS, and supernatants were harvested and analyzed for ELISA (eBioscience, San Diego, CA) according to the manufacturer's protocol.

### 2.8. Quantitative Real-Time PCR

Total RNA was isolated from the liver using RNAiso Plus (Takara) according to the manufacturer's protocol. One microgram total RNA was reverse transcribed to first-strand cDNA, which was performed with PrimeScript™ RT Master Mix (Takara), and then expression levels of mRNA were quantified by real-time PCR using SYBR Premix Ex Taq™ (Takara). The PCR program included 1 cycle of 95°C for 30 s and 40 cycles of 95°C for 5 s and 60°C for 30 s. The specific primers used for amplification were shown in Table 1. The results were expressed by calculating the 2^-ΔΔCT^ values and the housekeeping gene is GAPDH.

### 2.9. Flow Cytometry Analysis

The liver tissue was shredded, grinded by 200-mesh screen mesh, and filtered to a 50 ml centrifuge tube. To isolate mouse T cells, hepatocytes were prepared and other cells depleted using a mouse pan T cell isolation kit (Miltenyi Biotec) according to the manufacturer's instructions. For surface staining, the livers were collected and washed with PBS once, then incubated for 30 min with fluorochrome-conjugated antibodies as follows: PE-CD3, FITC-CD4, and PC5.5-CD8 (eBioscience). For intracellular staining, liver cells were stimulated with 100 ng/ml PMA (Sigma), 750 ng/ml ionomycin (Sigma), and 2 *μ*M monensin (BD Biosciences) for 5 h. The livers were collected and incubated with anti-CD4, then fixed, permeabilized, and stained with Mouse Th1/Th2/Th17 Phenotyping Cocktail (BD Biosciences) and FITC-Foxp3 (eBioscience). Flow cytometry analyses were performed on FACSCalibur (BD).

### 2.10. Statistical Analysis

The data were analyzed by GraphPad Prism software (GraphPad Software Inc., San Diego, CA). All quantitative data were expressed as mean ± SEM as indicated. The comparison between the two groups was analyzed by unpaired Student's *t*-test, and multiple comparisons were compared by one-way ANOVA followed by Dunnett's test. Statistical significance was established at *p* < 0.05.

## 3. Results

### 3.1. Koumine Protects the Rats from NAFLD

**Figure 2:**
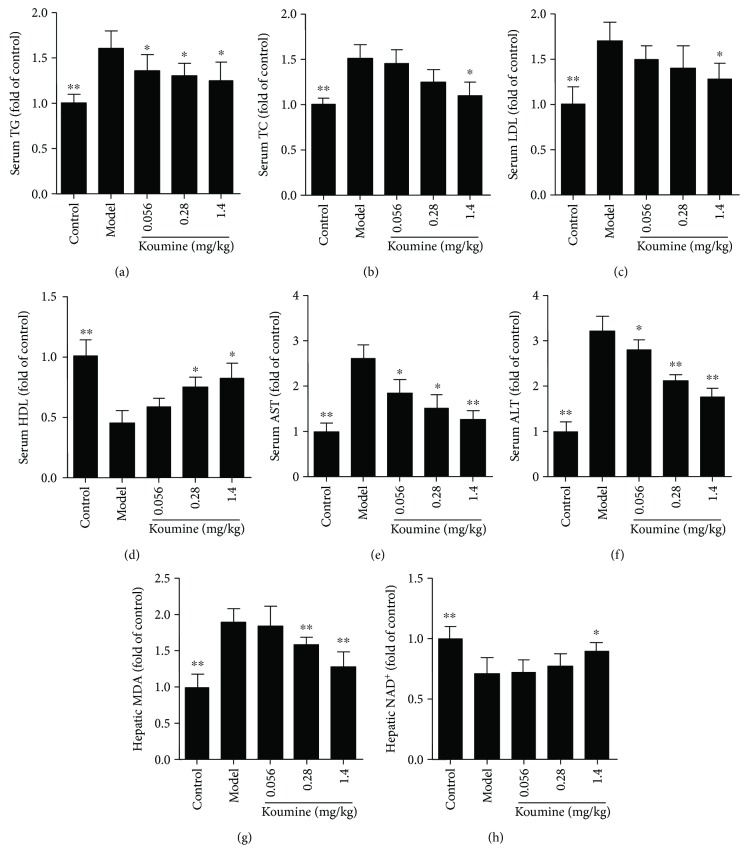
Effect of koumine on serological indexes of NAFLD rats induced by fat diet. TG: total cholesterol; TG: triglyceride; HDL: high-density lipoprotein; LDL: low-density lipoprotein; AST: aspartate aminotransferase; ALT: alanine aminotransferase; hepatic malondialdehyde (MDA) and nicotinamide adenine dinucleotide (NAD). ^∗^*p* < 0.05 and ^∗∗^*p* < 0.01, compared with the model group.

**Figure 3:**
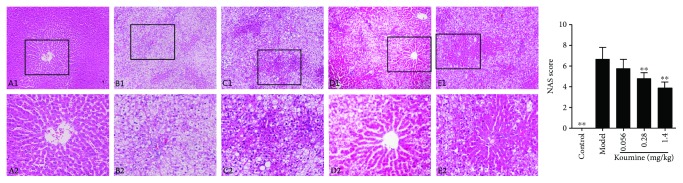
Effect of koumine on the pathomorphology of liver tissue induced by fat diet in NAFLD rats. A1-E1 represent the control group, model group, 0.056 mg/kg koumine-treated group, 0.28 mg/kg koumine-treated group, and 1.4 mg/kg koumine-treated group, respectively (×100 magnification); A2-E2 represent the same group with ×200 magnification. ^∗∗^*p* < 0.01 compared with the model group.

NAFLD is recognized to represent the hepatic manifestation of the metabolic syndrome. We asked whether administration of koumine could affect the disease progression of NAFLD. Compared with the control group, the liver index of the rats in the model group increased significantly. Koumine treatment at the doses of 0.28 and 1.4 mg/kg significantly decreased the liver index of the control group compared with that of the model group (Figure 1(b)). Furthermore, we investigated the effects of koumine on serological indexes of NAFLD rats induced by fat diet. TG, TC, LDL, ALT, AST, and MDA in the model rats were significantly increased, and the content of HDL and NAD was significantly decreased compared with the control group. The level of TG, TC, LDL, ALT, AST, and MDA in the koumine-treated rats was significantly lower than that in the model group. The content of HDL in serum and NAD in the liver of rats was higher than that in the model group and increased in a dose-dependent manner (Figure 2). Moreover, the effect of koumine on the histopathological morphology of the liver was investigated in NAFLD rats induced by fat diet. In the control group, the liver surface was smooth and the hepatic lobule structure was clear. There were no hepatocyte swelling, fatty lesions, and inflammatory cell infiltration in the portal area. In the model group, the surface of the rat liver was roughened by the yellow soil. The volume of the liver increased obviously, and the hepatocytes showed fatty degeneration with necrosis and inflammatory cell infiltration. After koumine treatment, different degrees of improvement were observed and the proportion of nonfat liver cells increased significantly (Figure 3).

### 3.2. Koumine Suppresses Proinflammatory Cytokine Production and Expression in NAFLD Rats

**Figure 4:**
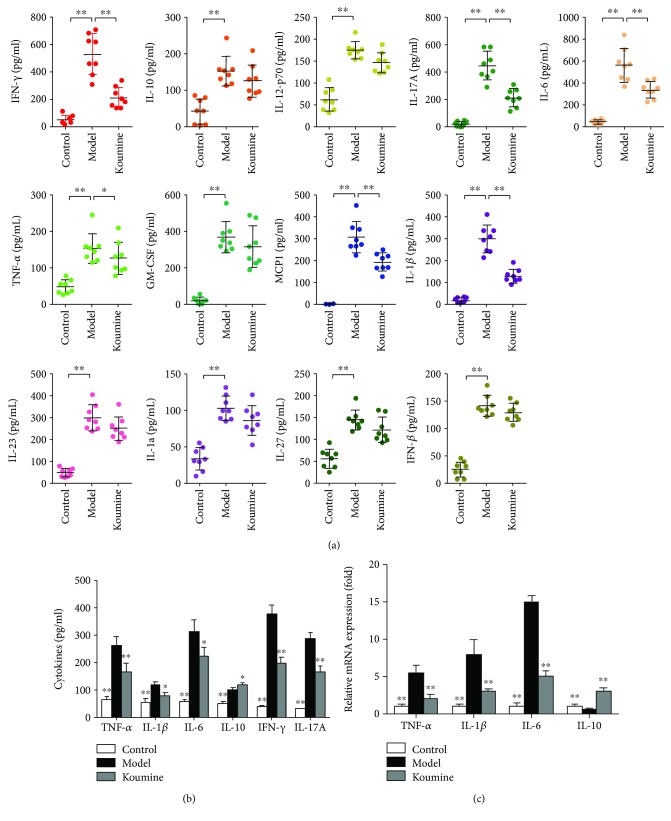
Koumine reduces the production and mRNA expression of cytokines in activated T cells. (a) Model and 1.4 mg/kg koumine-treated rats were sacrificed to obtain the serum and measured for the cytokines by multifactor detection of flow cytometry. (b) The livers were isolated from model and koumine-treated rats, and after being homogenized, the supernatants were harvested to measure the cytokine production by ELISA. (c) The livers were isolated from model and koumine-treated rats, and mRNA expression of selected genes were measured by real-time PCR. Data are means ± SEM (*n* = 3). ^∗^*p* < 0.05 and ^∗∗^*p* < 0.01 versus model. Data presented are representative of three independent experiments.

We next investigated the effect of koumine on serum levels of cytokines by multifactor detection of flow cytometry. Koumine administration significantly decreased the proinflammatory cytokines IFN-*γ*, IL-17A, TNF-*α*, IL-6, MCP1, and IL-1*β* levels in serum, while cytokines IL-10, IL-12, GMCSF, IL-23, IL-1*α*, IL-27, and IFN-*β* levels had a weakening trend (Figure 4(a)). Due to the noticeably preventive effect of koumine on NAFLD, we wondered whether koumine can suppress the inflammatory response. The liver isolated from model and koumine-treated rats were homogenized, and the supernatants were collected to analyze the production of proinflammatory cytokines. Compared with the model group, treatment with koumine significantly reduced the production of IL-6, IL-1*β*, IFN-*γ*, IL-17A, and TNF-*α*, while increasing the anti-inflammatory cytokine IL-10 level (Figure 4(b)). Subsequently, we investigated the effect of koumine on the mRNA expression of proinflammatory cytokines in the liver of different groups. The mRNA expression of IL-6, IL-1*β*, and TNF-*α* was also decreased while the anti-inflammatory cytokine IL-10 level was increased by koumine treatment (Figure 4(c)).

### 3.3. Koumine Increases CD4^+^ Cell Proportion in Liver Lymphocytes

**Figure 5:**
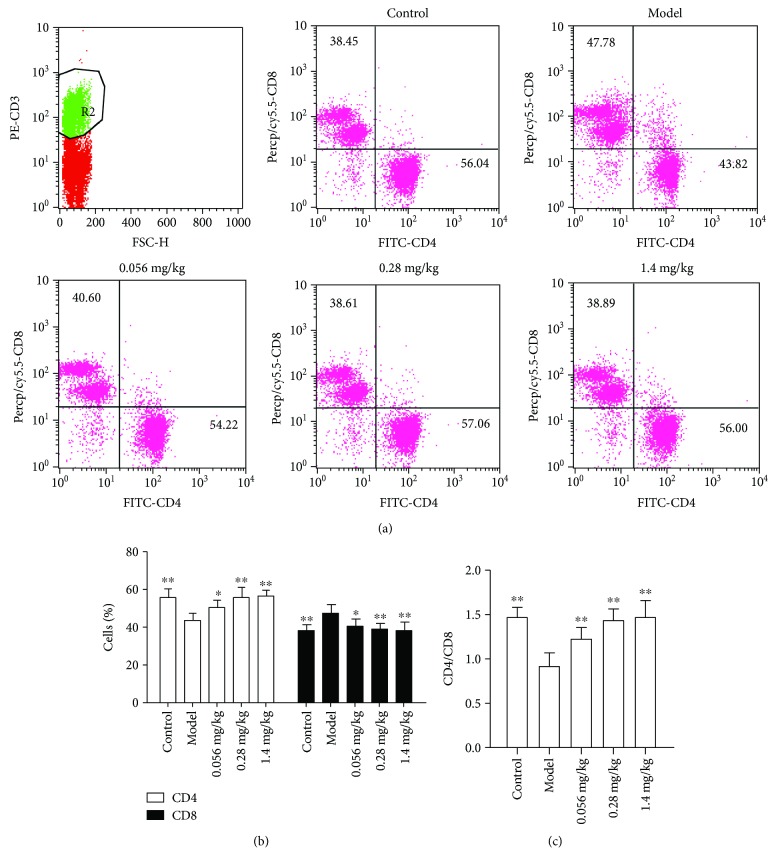
Koumine reduces the number of total leukocytes and CD4^+^ T cells infiltrated in the liver. The livers were isolated from model and koumine-treated rats. (a) Cells were analyzed for expression of CD4, CD8, and CD3 by flow cytometry. (b) The percentages of cells that are positive to these antigens were represented. ^∗^*p* < 0.05 and ^∗∗^*p* < 0.01 versus model.

The livers were isolated from model and koumine-treated rats at the end of disease. Cells were stained with anti-CD3, anti-CD4, and anti-CD8 to analyze the infiltration of total leukocytes, CD4^+^ T, and CD8^+^ T cells by flow cytometry. Compared with the model group, koumine can significantly increase CD4^+^ and decrease CD8^+^ cell percentage in liver lymphocytes (Figure 5).

### 3.4. Koumine Inhibits Th1 and Th17 while Promoting Th2 and Treg in Liver Lymphocytes

**Figure 6:**
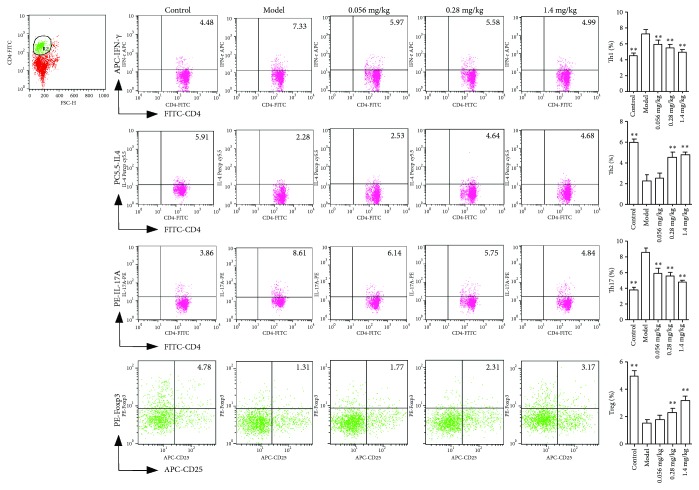
Koumine suppresses the differentiation of Th1 and Th17 cells while enhances Th2 and Treg cells. Cells were analyzed for the percentages of Th1, Th2, Th17, and Treg cells in the CD4 subsets by flow cytometry. ^∗∗^*p* < 0.01 versus the model group.

As we all know, activated T cells can be differentiated into a variety of subtypes, including Th1, Th2, Th17, and Treg cells, and the imbalance of subtype differentiation is closely related to the progress of disease. To account for the abovementioned results, koumine significantly increased the number of CD4^+^ T cells infiltrated into the liver, so it is necessary to investigate the effect of koumine on the subtypes of CD4^+^ T cells. First of all, liver monocytes isolated from naive, NAFLD, and koumine-treated rats were analyzed for the Th1 (CD4^+^IFN-*γ*^+^), Th2 (CD4^+^IL-4^+^), Th17 (CD4^+^IL-17^+^), and Treg (CD4^+^Foxp3^+^) cell populations by flow cytometry. Compared with naive rats, the NAFLD rats showed a significantly increased number of Th1 and Th17 and decreased Th2 and Treg, while koumine could reverse the trends in contrast to the model group (Figure 6).

## 4. Discussion

The liver is recognized as an immunological organ playing critical roles in fighting invading pathogens. It hosts hepatocytes and nonparenchymal cells. The nonparenchymal cells include large populations of immune cells such as lymphocytes which have complex interactions with each other and with the hepatocytes, making the liver an important organ bridging innate immunity and adaptive immunity. The dysregulation of immune cells in the liver is involved in almost all types of liver diseases, such as NAFLD [25, 27]. Currently, effective treatment for inflammatory liver diseases such as NAFLD remains limited [28, 29]. An improved understanding of the inflammatory processes responsible for the progression of liver diseases will be very helpful in developing novel and effective strategies for NAFLD treatment [30]. In the present study, treatment with koumine effectively inhibited the development of NAFLD and reduced the serum proinflammatory cytokine levels. Koumine-treated rats displayed less leukocytes and CD4^+^ T cells aggregated in the liver. With regard to CD4^+^ T cells, we demonstrated that koumine decreased the percentages of CD4^+^IFN-*γ*^+^ and CD4^+^IL-17^+^ cells in the liver. Furthermore, koumine reduced the production and mRNA expression of proinflammatory cytokines *in vivo*.

The pathogenesis of NAFLD is not clear. It is thought that the pathogenesis of NAFLD is related to immunity and inflammation. CD4^+^ T cells play a crucial role in metabolic syndrome. The diverse functions of CD4^+^ T cells depend on their multiple subtypes. Activated CD4^+^ T cells can be differentiated into at least Th1, Th2, Th17, and Treg. Among them, Th1 and Th17 cells always play a pathogenic role, yet Th2 cells display an antagonistic function to Th1 and Treg cells undertake the responsibility of regulating the immune responses [31, 32]. To mention the pathogenesis of NAFLD, the effector cells promote the activated CD4^+^ T cell differentiation into more Th1 and Th17 subtypes and less Th2 and Treg subtypes; subsequently, Th1 and Th17 cells migrate across the liver [33]. As demonstrated above, we comprehended that the effect of koumine may be targeted on CD4^+^ T cells; thus, we isolated the liver from naive, model, and koumine-treated rats to further investigate the effect of koumine on the differentiation of CD4^+^ T cells. The result showed that koumine-treated rats presented decreasing trends for the percentages of Th1 and Th17 cells and increasing Th2 and Treg cells compared to model rats. So we demonstrated that koumine ameliorated NAFLD through modulating the proportion of different subtypes of helper T cells. Otherwise, we future investigated the related cytokines that participate in controlling the differentiation of CD4^+^ T cells. The present experiments showed that koumine could inhibit the immune response and the secretion of Th1- and Th17-type cytokines in CD4^+^ T lymphocytes, increase the level of Th2 type cytokines, and regulate the cytokine network, suggesting that koumine has immunosuppressive and anti-inflammatory effects, and the anti-NAFLD effect of koumine may also be mediated by its immunomodulatory action.

NAFLD is a chronic inflammatory process, with a number of proinflammatory cytokines released by activated immune cells in the peripheral immune organs [34]. Previous studies have shown that inflammatory cytokines such as TNF-*α*, IL-6, and IL-1*β* are predominantly detected in NAFLD mice [35]. TNFR1 signaling is considerable in the development of demyelination and contributes to the limitation of T cell responses during immune-mediated CNS disease [36]. The high level of IL-6 mRNA expression in the peripheral immune organs correlates with the progression of NAFLD. IL-6 and TNF-*α* are also the important cytokines secreted by Th17 and Th1 cells [37]. We observed that koumine suppresses the production of IL-6, IL-1*β*, and TNF-*α* along with their expression of mRNA levels in the liver, indicating that koumine prevents the onset of NAFLD also through the inhibition of proinflammatory cytokines in the peripheral immune organs.

In summary, our study demonstrates that koumine ameliorates NAFLD with an appropriate tolerance. Koumine further reduced the Th1 and Th17 cells and increased Th2 and Treg cells *in vivo*. Furthermore, koumine also reduces the production of related cytokines in the liver, indicating that it ameliorates NAFLD mainly through modulating the proportion of different subtypes of helper T cells and weakens the immune responses. However, this study is far from enough to explain the effect of koumine, and further investigations should be performed to elucidate the penetrating mechanisms that koumine acts and discover the possible effects on other metabolic diseases.

## Figures and Tables

**Table 1:** The specific primers used for amplification.

Gene	Forward 5′-3′	Reverse 5′-3′
TNF-*α*	CACCACCATCAAGGACTCAA	GAGACAGAGGCAACCTGACC
IL-6	TTCTTGGGACTGATGCTG	CTGGCTTTGTCTTTCTTGTT
IL-10	GGAAGAGAAACCAGGGAGAT	GCAGACAAACAATACACCATTC
IL-1*β*	GCCCATCCTCTGTGACTCAT	AGGCCACAGGTATTTTGTCG
GAPDH	AGTGGCAAAGTGGAGATT	GTGGAGTCATACTGGAACA

## Data Availability

The data used to support the findings of this study are available from the corresponding author upon request.
